# Perceptions of police legitimacy and its influencing factors: evidence from Guangzhou, China

**DOI:** 10.3389/fpsyg.2026.1903028

**Published:** 2026-10-26

**Authors:** Huijia Lu, Senlin Zheng, Jiaxin Zheng, Guangwen Song, Luzi Xiao

**Affiliations:** 1 School of Law, South China University of Technology, Guangzhou, Guangdong, China; 2 Center of GeoInformatics for Public Security, School of Geography and Remote Sensing, Guangzhou University, Guangzhou, Guangdong, China

**Keywords:** perceptions of police legitimacy, procedural justice, police performance evaluations, environmental characteristics, Guangzhou

## Abstract

**Introduction:**

Understanding the foundations of public trust in law enforcement is critical for effective governance, particularly in non-Western contexts. While procedural justice theory has received growing empirical attention in China, existing studies have produced mixed findings and largely neglected the combined influence of interactional experiences, neighborhood environmental conditions, and institutionally embedded factors on police legitimacy perceptions.

**Methods:**

This study examines perceptions of police legitimacy in Guangzhou using survey data from 1,899 residents across 72 diverse neighborhoods. The factors shaping legitimacy perceptions are categorized into five broad domains: individual attributes, procedural/distributive justice, police performance evaluations, legal socialization experience, and perceptions of community environment. Ordinary least squares regression models were estimated to assess the relative contribution of each domain.

**Results:**

Police performance evaluations were most strongly associated with perceived legitimacy, with police work capability emerging as the strongest positive correlate, followed by overall satisfaction with police and perceived effectiveness of police stops. Procedural and distributive justice also exerted significant but comparatively weaker influence, while legal socialization experiences --particularly prior stops and stricter legal attitudes --were associated with lower perceptions of legitimacy. At the individual level, local hukou holders reported higher perceptions of legitimacy, whereas married individuals and those in higher-status neighborhoods unexpectedly reported lower ratings. Regarding environmental characteristics, community cohesion was associated with higher perceptions of police legitimacy.

**Discussion:**

These results complement and extend existing models by highlighting the contextual specificity of legitimacy formation in China’s urban setting. Our findings underscore the need for policing strategies that prioritize routine, high-quality citizen interactions and community engagement.

## 1 Introduction

Understanding the foundations of public trust in law enforcement is a central concern for criminology and public policy. A robust body of research, largely grounded in Tyler’s process-based model of legal socialization, establishes that citizens’ perceptions of police legitimacy are primarily shaped by their experiences of procedural justice—being treated with fairness, respect, and voice during encounters with authorities (Tyler, 2006; Fine et al., 2022; Li et al., 2024).

While this theoretical framework has been widely validated, recent scholarship calls for a more nuanced understanding of its application across different populations and contexts. A growing number of studies highlight that the determinants of police legitimacy can vary significantly based on age, life experience, and social setting. For instance, a recent scoping review on youth found that their judgments of police legitimacy are not only influenced by direct or vicarious contact with officers but are also deeply mediated by relationships with other authority figures (Li et al., 2024). As for social context, in the United States and the United Kingdom, procedural justice consistently emerges as a central correlate of legitimacy and of willingness to cooperate with the police (Sunshine and Tyler, 2003; Bradford et al., 2025). Studies conducted elsewhere qualify this picture. In Brazil, Oliveira (2025) finds that legal cynicism and exposure to intrusive policing shape legitimacy judgments in ways the process-based model does not anticipate. In Nigeria, Akinlabi and Murphy (2018) show that compliance with the law may be sustained by habit or fear rather than by normative obligation—what they call dull compulsion—which raises the question of what, if anything, legitimacy adds beyond obedience. In South Korea, Na et al. (2023) report that instrumental effectiveness and procedural justice operate as complementary rather than competing public values, which challenges the assumption that normative and instrumental motives are alternatives. This suggests that legitimacy is not formed in a vacuum but is embedded within a complex web of individual, interactional, and environmental factors.

Furthermore, there is a pronounced geographical imbalance in the evidence base. The vast majority of studies on police legitimacy have been conducted in Western societies. Consequently, our understanding of how these processes operate in the distinct urban landscapes of major non-Western cities, such as those in China, remains limited. The institutional structures, patterns of community organization, and forms of police–public engagement in these settings may give rise to unique configurations of legitimacy that are not fully captured by existing models. For example, Sun et al. (2013) found that Chinese citizens tend to conflate procedural- and outcome-based trust, and that expressive concerns—community trust and quality of life—outweigh instrumental concerns in shaping police trust among migrants, villagers, and urbanites. Zhang et al. (2020) demonstrated that confidence varies across police agency levels in Shanghai, reflecting a hierarchical structure requiring differentiation between national, provincial, and local police. Zhao et al. (2014) found that objective disorder crime did not directly affect perceptions of police performance, highlighting the gap between objective conditions and subjective evaluations. Wang and Sun (2020) further reveals that with urban residents exhibiting lower outcome-based trust than their rural counterparts.

Although prior literature has increasingly attended to background variables such as race, socioeconomic status, and other individual characteristics, as well as police–citizen relational dynamics and neighborhood conditions, these factors have largely been examined in isolation (Weitzer and Tuch, 1999; Tyler, 2003; Brunson, 2007; Gau et al., 2012; Zhang et al., 2020). In sum, there are two gaps in current study about perceptions of police legitimacy. First, the question of relative importance—namely, which set of factors exerts the strongest influence on legitimacy perceptions—remains unresolved, especially in non-Western contexts (Sun et al., 2018; Cao, 2022), and systematic comparisons of competing mechanisms are still scarce. Second, the evidence base remains geographically imbalanced, with limited empirical attention to Chinese megacities that are distinguished by unique institutional arrangements (e.g., the hukou system) and community structures.

To address this lacuna, our study draws on original survey data from 72 diverse communities in Guangzhou, one of China’s most prosperous and populous megacities. We construct a comprehensive five-dimensional analytical framework that integrates individual attributes, procedural/distributive justice, police performance evaluations, legal socialization experience, and residents’ perceptions of community environment. By doing so, we aim to provide a more holistic and contextually grounded account of the factors that shape public perceptions of police legitimacy, contributing to a more universally applicable theory of legal authority.

## 2 Theories and prior findings

### 2.1 Measuring perceptions of police legitimacy

Legitimacy denotes the perceived rightfulness of authority, institutions, or rules that elicits voluntary deference, compliance, and cooperation—distinct from coercion, self-interest, or fear of punishment (Tyler, 2006). In the policing context, it specifically refers to the public’s recognition of police as appropriate and proper authorities, accompanied by an internalized moral obligation to obey their directives because “it is the right thing to do,” with key foundations including procedural justice, perceived lawfulness, and normative obligation (Tyler, 2006; Hawdon and Tech, 2008).

Investigating citizens’ subjective perceptions of police legitimacy is more feasible than measuring the objective legality of police enforcement (Güss et al., 2020; Schade and Thielgen, 2022). This study examines the former: residents’ beliefs about whether the police are entitled to be obeyed and whether their authority is morally appropriate. Existing literature has employed methods such as questionnaire surveys, face-to-face or telephone interviews. Most studies employed Likert scales with multi-item indices for measurement. Generally, legitimacy has been measured in research through several key approaches: perceived obligation to obey, which assesses the duty to follow laws and legal directives even when disagreeing; support, allegiance, and trust, using indicators like respect for authorities and belief in their honesty; affective “thermometer” ratings, which quantify positive or negative feelings toward legal institutions on a numerical scale; and lack of cynicism about the law, a more recent framework that measures the degree to which people believe legal authorities act in their own interests rather than those of the powerful. Together, these methods capture the multifaceted nature of legitimacy, spanning behavioral obligation, emotional trust, and perceived fairness (Tyler, 2006).

The four-dimensional model proposed by Bottoms and Tankebe (2012) represents one of the most widely accepted frameworks currently in use. This model posits that the dimensions of procedural justice and distributive justice, together with perceived lawfulness and felt obligation to obey, collectively constitute the four components of perceptions of police legitimacy itself. The questionnaire items they employed to measure procedural justice and distributive justice were also broadly comparable to those used in Tyler’s research (Bottoms and Tankebe, 2012; Tankebe, 2013). Sun et al. (2018) used two measures of legitimacy—‘When police deal with people in my community, they always act according to the law’ and ‘The law represents the moral values of people like me’ – simultaneously examine residents’ assessments of the legitimacy of police enforcement and their own attitudes toward the law in China. In the recent study of Reisig et al. (2023), they empirically validated Pósch et al. (2021)’s normative obligation to obey the police scale, confirming it as a distinct construct separate from coercion-driven non-normative obligation. This work supports the scale’s utility for refining police legitimacy measurement by distinguishing normative motives for obedience from mere compliance or “dull compulsion”—the idea that people obey the law out of habit or fear rather than moral conviction (Akinlabi and Murphy, 2018).

### 2.2 Factors affecting the police legitimacy

#### 2.2.1 Procedural justice and distributive justice

Procedural justice, pioneered by Thibaut et al. (1974) and extended by Tyler, emphasizes that individuals evaluate fairness not solely based on outcomes but also on the fairness of decision-making processes (Thibaut et al., 1974; Tyler, 2003; Oliveira, 2025). A strong academic consensus holds that procedural justice is the primary driver of police legitimacy (Sunshine and Tyler, 2003; Reisig et al., 2007; Tankebe, 2013). It comprises two dimensions: quality of interpersonal treatment and quality of decision-making (Tyler, 2003; Bottoms and Tankebe, 2012; Tankebe, 2013). When citizens perceive dignity, respect, and fact-based decision-making from officers, their recognition of police authority deepens, fostering an internalized obligation to comply.

Distributive justice, by contrast, concerns the perceived fairness of policing outcomes—whether police services are equitably distributed across groups (Sunshine and Tyler, 2003). While distributive fairness positively influences legitimacy, its effect is generally weaker than procedural justice; outcome favorability and expectancy alignment exert negligible influence (Reisig et al., 2007). In the Chinese context, Sun et al. (2018) found that “police lawfulness” carried the largest factor loading in a structural equation model of legitimacy, followed by distributive justice, procedural justice, and police effectiveness—suggesting a nuanced hierarchy of influences.

#### 2.2.2 Police performance evaluations

Police performance evaluations are a set of pivotal determinants of residents’ perceptions of police legitimacy, encompassing diverse interactional dimensions and contextual factors (Rosenbaum, 2025). It is important at the outset to distinguish conceptually between performance evaluations and legitimacy itself. Performance evaluations are evaluative judgments concerning what police do—their effectiveness, responsiveness, visibility, and the quality of their service delivery. Legitimacy, by contrast, is a normative judgment concerning police authority—whether citizens believe the police have the right to be obeyed and whether the institution aligns with their own moral values (Tyler, 2006; Bottoms and Tankebe, 2012). This distinction implies that the two constructs are conceptually separable: citizens may regard the police as competent yet withhold normative deference, or conversely, may question police effectiveness while still recognizing the institution’s rightful authority. Performance evaluations are therefore best understood as antecedents of legitimacy rather than as constituent indicators of it (Reisig et al., 2007; Tankebe, 2013).

A central debate in the literature concerns the relative weight of instrumental versus procedural considerations in shaping legitimacy judgments. Instrumental concerns center on police effectiveness in fighting crime, sufficient presence, and quick responses—what policing scholars term the what of police work—whereas procedural justice concerns the how of police-resident interactions. While Tyler’s process-based model has long held that procedural fairness is the primary driver of legitimacy, a growing body of research demonstrates that performance evaluations exert significant independent effects (Sunshine and Tyler, 2003; Na et al., 2023).

The association between performance evaluations and legitimacy is more nuanced than a simple bivariate relationship. Bradford et al. (2025) demonstrate that different aspects of police performance have distinct associations with public attitudes: visibility and presence are more important for overall confidence, while fairness and proportionality are more strongly associated with legitimacy judgments. In the Korean context, Na et al. (2023) found that perceptions of police effectiveness and procedural justice are both positively associated with trust in police, challenging the dichotomy that positions instrumental and normative motives as competing rather than complementary.

The nature and valence of police–citizen encounters also exert differential effects on legitimacy. Brunson and Miller (2006) find that police corruption exerts a significantly negative impact on police legitimacy, whilst negative, involuntary encounters—such as harassment, abuse, or violence—directly diminish residents’ evaluations of police legitimacy.

#### 2.2.3 Legal socialization experience

Legal socialization refers to the lifelong process through which individuals acquire values, attitudes, and orientations toward the law, legal authorities, and the legal system. This process unfolds across the life course, with adolescence representing a particularly formative period during which foundational frameworks for evaluating legal authority are constructed. Fagan and Tyler (2005)identify three core domains of legal socialization: legal cynicism, legitimacy, and moral disengagement. Complementing this perspective, Cohn et al. (2012) conceptualize legal socialization as the internalization of codified societal norms, wherein individuals’ beliefs about rules and rule-violation are shaped through interactions with various authority figures.

Empirical studies have substantiated this broader view. Rosenbaum et al. (2005), in a longitudinal study of Chicago residents across different racial groups, found that direct contact alone does not necessarily alter attitudes. Rather, negative citizen-initiated encounters (as opposed to police-initiated ones) and vicarious experiences—whether positive or negative—significantly influence attitude changes. Notably, individuals who had never interacted with the police tended to hold neutral or mildly positive views toward them (Frank et al., 2005; Wolfe, 2011; Debbaut and De Kimpe, 2023). Extending this line of inquiry to rural settings, Adorjan et al. (2017) similarly found that informal and transparent contacts fostered favorable perceptions among rural Canadian youth, whereas delayed responses and formal law-enforcement framing eroded trust. Their findings further highlight that positive informal contacts, such as police officers acting as community members or coaches, foster favorable perceptions, whereas delayed responses, formal law enforcement framing, and lack of transparency erode trust—underscoring the need for context-specific policing strategies tailored to rural environments.

#### 2.2.4 Individual attributes

Existing studies indicate that individual demographic and socioeconomic attributes shape residents’ perceptions of police legitimacy, though findings often vary by context.

Gender and Age. The literature on gender effects remains inconclusive. Most studies report no significant direct effect of gender on legitimacy perceptions (Weitzer, 1999; Triplett et al., 2005; Wolfe, 2011; Gau et al., 2012; Fine et al., 2022), but some research suggests that men rate legitimacy lower due to harsher police treatment rooted in gendered stereotypes (Hawdon, 2003; Brunson and Miller, 2006; Lee et al., 2010; Cohn et al., 2012), while more recent work indicates that women’s evaluations may now be slightly lower as their rights consciousness grows (Debbaut and De Kimpe, 2023). Age typically correlates negatively with legitimacy: older adults, with greater social experience and exposure to institutional interactions, tend to be more sensitive to policing deficiencies. Nevertheless, this effect appears negligible in some economically declining mid-sized cities (Hinds, 2009; Lee et al., 2010; Cohn et al., 2012; Gau et al., 2012; Trinkner et al., 2019; Fine et al., 2022).

Marital Status and Education. Marital status has received limited empirical attention. Scaglion and Condon (1980) suggest that separated or divorced individuals may hold more negative views due to heightened emotional sensitivity in police-related conflicts and weakened social networks. Educational attainment presents a “legitimacy paradox”: higher education fosters critical thinking about procedural injustices, lowering legitimacy ratings (Weitzer and Tuch, 1999; Hawdon, 2003; Tyler, 2003; Triplett et al., 2005). However, some studies examining residents’ attitudes toward the police or their willingness to cooperate have found that education exerts a significant positive influence on both satisfaction with the police and willingness to cooperate (Weitzer, 1999; Weitzer and Tuch, 2005).

Income, Socioeconomic Status, and Language. Personal income generally shows no direct effect on legitimacy perceptions (Scaglion and Condon, 1980; Weitzer, 1999; Weitzer and Tuch, 1999; Tyler, 2003). By contrast, perceived community socioeconomic status correlates positively with legitimacy, as middle-class neighborhoods report less police misconduct (Weitzer, 1999; Brunson, 2007; Gau et al., 2012). More broadly, Cao (2022) critically examined police legitimacy in stratified democracies, arguing that race/ethnicity remains understudied and legitimacy must be understood within democratic principles and social stratification. Language barriers may impede citizen participation and interpretation, thereby affecting perceptions of decision-making neutrality. Tyler (2003) has suggested that non-English speakers may feel disrespected or suffer diminished dignity if police fail to provide linguistic assistance. Language barriers may prevent citizens from comprehending the rationale behind police actions or fully grasping the motivations behind law enforcement, thereby diminishing trust in the police.

Hukou Status and Police Legitimacy. The hukou system—China’s household registration policy—stratifies urban populations into local residents (bendi renkou) and non-local residents (wailai renkou), with profound implications for access to social welfare, public services, and institutional trust (Wang and Sun, 2020). In major metropolitan areas, non-local residents are predominantly migrant workers from rural or less-developed regions, whereas local residents typically comprise urbanites and villagers with full entitlement to municipal benefits (Wu et al., 2016).

A substantial body of literature suggests that non-local residents tend to exhibit lower trust in the police compared to their local counterparts, with several explanations proposed for this disparity. First, migrants are disproportionately associated with higher crime rates in public discourse, rendering them more susceptible to police surveillance and stops (Sun et al., 2013). Second, migrant workers may encounter procedural unfairness during police encounters—ranging from arbitrary identity checks to unequal enforcement of regulations (Cao, 2022). Third, limited access to local welfare and social integration may foster a sense of marginalization that generalizes into diminished trust in state authorities, including the police (Wang and Sun, 2020).

However, this pattern is not uniform across contexts. Sun et al. (2013), analyzing survey data from three coastal cities in China, found that migrant workers actually reported higher levels of police endorsement than both local villagers and urban residents—a counterintuitive finding that the authors attributed to migrants’ relative satisfaction with urban opportunities compared to their rural origins, as well as their lower expectations of police service. Similarly, Wang and Sun (2020) examined urban–rural differences in Taiwan and found that while residents differed significantly on outcome-based trust measures, no such gap emerged in procedural-based trust, suggesting that the hukou-trust relationship may be more nuanced than previously assumed. More recently, Wu et al. (2016) using nationwide data from the Chinese General Social Survey, found that rural hukou holders actually held more favorable views of the police than their urban counterparts, with self-identified social status emerging as the most important stratifying variable explaining trust in the police. These mixed findings suggest that the effect of hukou on police legitimacy is not deterministic but mediated by individual expectations, perceived social status, and the specific dimension of trust under examination.

#### 2.2.5 Environmental characteristics

Liu et al. (2020) conducted a multilevel study using survey data from Shanghai, China, to explore how neighborhood and individual factors shape residents’ willingness to help the police, finding that neighborhood collective efficacy is a significant positive predictor, while police legitimacy measures (e.g., obligation to obey, trust) and their antecedents (fairness, effectiveness) are non-significant after controlling for neighborhood characteristics. The study also revealed that police presence is positively associated with residents’ willingness to assist the police, highlighting the relevance of community cohesion and visible policing in the Chinese context. Contrary to collective efficacy, social disorder represents the concentration of socio-economic issues within a community, such as poverty, unemployment, welfare dependency, and single-parent households. It serves as a core macro-level variable for measuring the fragility of a community’s social structure. The greater the social disadvantage, the weaker the evaluation of police legitimacy, exhibiting a significant negative impact (Triplett et al., 2005; Brunson and Miller, 2006; Stewart et al., 2009; Gau et al., 2012). Further, police typically prioritize areas with severe physical disorder, which are often accompanied by high crime rates (Chen and Rafail, 2022). However, whether physical disorder influences perceptions of police legitimacy remain to be examined.

#### 2.2.6 Analytical framework and current focus

The foregoing review reveals two gaps in the literature on perceptions of police legitimacy. First, the question of relative importance—which set of factors most strongly shapes legitimacy perceptions—remains unresolved, particularly in non-Western contexts (Sun et al., 2018; Cao, 2022). Yet systematic comparisons of competing mechanisms remain scarce. Second, the evidence base remains geographically imbalanced, with limited empirical scrutiny of Chinese megacities characterized by distinct institutional arrangements (e.g., the hukou system) and community structures.

To address these gaps, this study constructs a five-dimensional analytical framework that integrates: (1) individual attributes; (2) procedural/distributive justice; (3) police performance evaluations; (4) legal socialization experience; and (5) perceptions of community environment. By situating these dimensions within a single model, we pursue three objectives: (a) to assess the relative contribution of each dimension to legitimacy perceptions; (b) to examine the roles of context-specific factors—particularly hukou and neighborhood social organization; and (c) to provide empirically grounded insights for policing policy in non-Western megacities. The analytical framework is shown in Figure 1.

**Figure 1:**
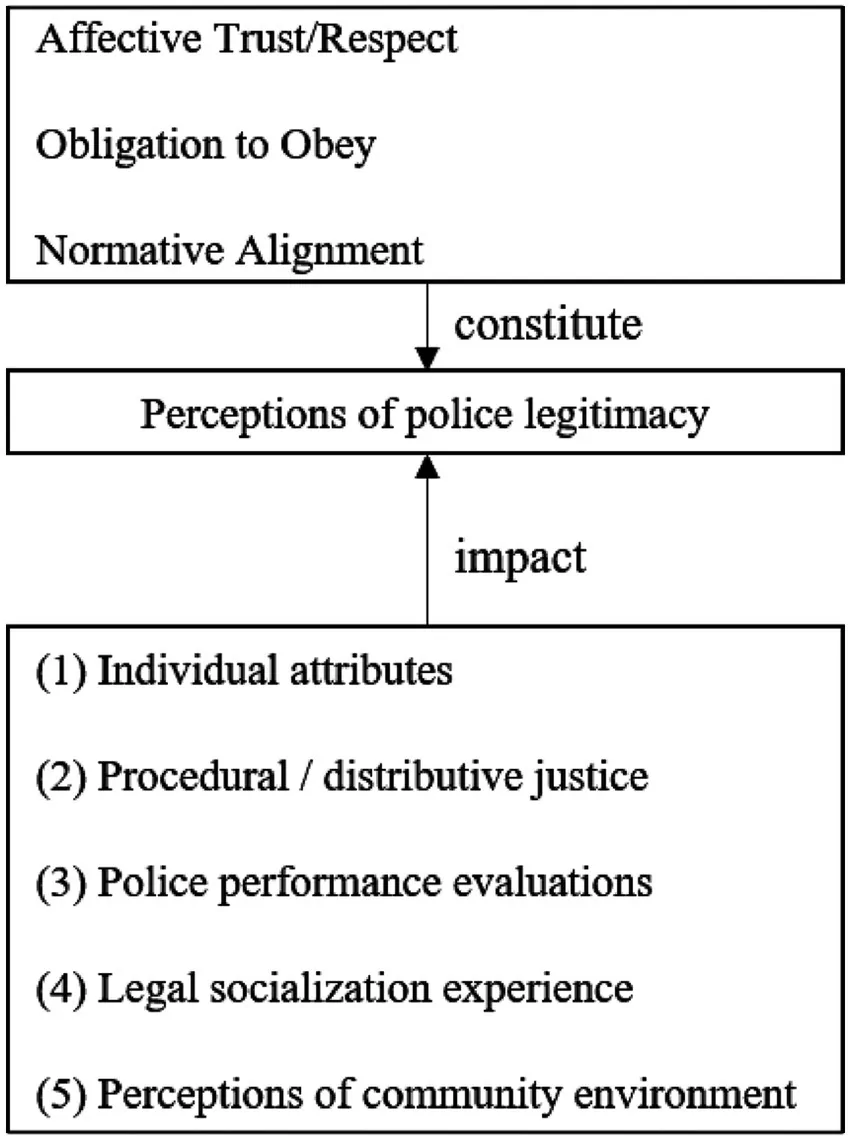
Analytical framework of perception of police legitimacy.

## 3 Data and methods

### 3.1 Data

This study focuses on Guangzhou, one of China’s largest metropolitan areas, covering 7,434 km^2^ and hosting a population of 18.978 million (as of 2024). Among the residents, nearly half are migrants without Guangzhou household registration (hukou). As a densely populated, highly mobile, and extensively urbanized metropolis, Guangzhou presents considerable challenges in urban governance.

The questionnaire survey was conducted in 72 communities (shequ) among the total of 2,775 communities, ranging between November and December 2024. The stratified sampling method was implied in selecting the 72 communities. Firstly, 2,775 communities in Guangzhou was clustered into 7 categories based on the 7th census. They are social housing communities, middle-income communities, university communities, suburban ageing communities, central ageing communities, central migrant communities and suburban migrant communities, respectively. Secondly, by incorporating the spatial distribution of different community types and findings from field investigations, 72 representative communities were proportionally selected from seven categories within the urban area (Figure 2). Thirdly, in each selected community, questionnaires were also allocated proportionally based on age, gender and their Hukou status. Then, two rounds of face-to-face surveys were conducted. In the first round, 1,944 questionnaires were distributed, of which 89 invalid responses were excluded, resulting in a validity rate of 95.4%. In the second round, supplementary surveys were subsequently carried out in selected communities, yielding 44 valid paper questionnaires. Ultimately, 1,899 valid questionnaires were retained. The specific questionnaire items used in this paper are detailed in Appendix A.

**Figure 2:**
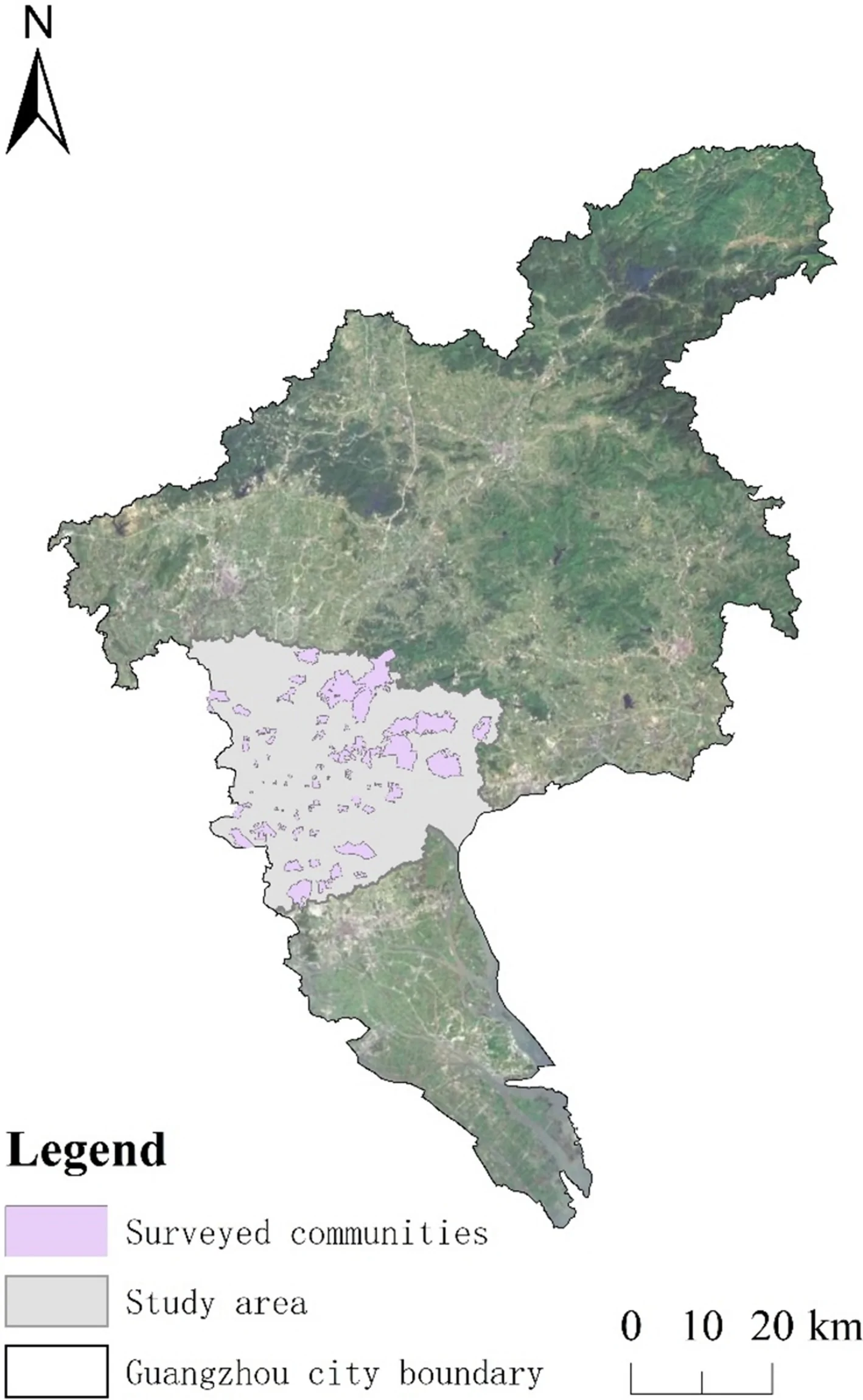
72 surveyed communities of Guangzhou.

### 3.2 Measures

#### 3.2.1 Dependent variable: perceptions of police legitimacy

The dependent variable in this study is perceptions of police legitimacy. Grounded in prior research on the measurement of public perceptions (Sunshine and Tyler, 2003; Tyler, 2003; Tyler, 2006; Wolfe, 2011), we operationalize police legitimacy using four items that capture both the normative and affective dimensions of legitimacy: E141 (“I hold the police in high regard”) taps affective trust/respect toward police authority, a dimension consistently used in legitimacy research (Sunshine and Tyler, 2003). If the public does not trust the police, the police can hardly be regarded as legitimate. E142 (“People should support the work of the police”) and E143 (“I would comply with police decisions during law enforcement even if I did not fully agree with them”) directly capture the felt obligation to obey dimension, widely recognized as the core behavioral component of legitimacy (Tyler, 2006; Reisig et al., 2007); and E144 (“The sense of right and wrong upheld by the police in handling cases aligns with my own values”) reflects normative alignment or moral congruence between citizens and police, a dimension that Bottoms and Tankebe (2012) identify as central to legitimacy judgments. This multi-dimensional approach allows us to distinguish legitimacy from conceptually related constructs such as trust, confidence, and satisfaction, which focus more on performance evaluations than on moral obligation. Each item is rated on a 5-point Likert scale ranging from 1 (“Strongly disagree”) to 5 (“Strongly agree”). Higher scores reflect stronger public perceptions of police legitimacy. The mean scores for the four items were 4.18, 4.24, 3.77, and 3.82, respectively, yielding an overall mean of 4.00 (range: 1–5).

#### 3.2.2 Independent variables

The independent variables are categorized into 5 groups: individual attributes, procedural/distributive justice, police performance evaluations, legal socialization experience, and perceptions of community environment.

##### 3.2.2.1 Individual attributes

Drawing on prior literature, this study incorporates nine individual attributes: gender, age, marital status, household registration status (local = 1), educational attainment, personal monthly income, length of residence, perceived community socioeconomic status, and Cantonese proficiency.

According to item A1, gender was coded as a binary variable (1 = female, 0 = male). Among all the interviewees, there are 898 females and 1,001 males. 47.29% of them are females and 52.71% are males. As for age (item A2), respondents were limited to those aged 20 and above, with age accordingly measured as a continuous variable from 20 years upward. We calculated participants’ ages by surveying their birth dates, subtracting the year of birth from the year of the interview conducted in 2024.

Item A3 concerning marital status (married = 1) offered three options: unmarried, married, and divorced or widowed. Married individuals were recorded as 1, while others were recorded as 0. Among these, 70.67% were married, with the remaining 29.33% comprising unmarried, divorced, or widowed individuals.

Household registration status (hukou) data is derived from item A4, disaggregated to the municipal level of administrative division. Respondents holding household registration in Guangzhou are classified as local residents and assigned a score of 1. Our statistical analysis reveals that 43.50% of the residents interviewed hold Guangzhou household registration, while the remaining 56.50% are non-local residents that holding non-Guangzhou household registration.

Educational attainment was assessed through item A5, which asked respondents to indicate their highest level of education on an ordinal scale from 1 (“No formal education”) to 8 (“Postgraduate degree or higher”), coded such that higher values indicate more advanced education. The mean score was 4.836 (SD = 1.624), with a range of 2–8.

Personal monthly income was measured using item A8, which asked respondents to select from eight ordered categories ranging from 1 (“Under ¥1,000”) to 8 (“Over ¥20,000”), with higher scores indicating higher income levels. The mean score was 4.599 (SD = 1.569), with a range of 1–8.

Length of residence was derived from item A11 (“When did you start living in this community (shequ)?”), computed by subtracting the reported year of move-in from the survey year (2024). Length of residence averaged 13.102 years, with a range of 1–81 years.

Perceived community socioeconomic status was measured using item A14, which asked respondents to rate their community’s standing in Guangzhou on a 5-point scale from 1 (“Low”) to 5 (“High”). The mean score was 3.162 (SD = 0.788).

Cantonese proficiency was assessed via item A17: “Can you speak Cantonese?” with options from (1) “Not at all” to (5) “Very fluent,” coded on a 1–5 scale reflecting increasing proficiency. The mean score was 3.887 (SD = 1.366).

##### 3.2.2.2 Procedural/distributive justice

Procedural justice and distributive justice were operationalized using two specific variables: procedural justice (items E11-1, E11-2) and distributive justice (item E11-3).

Procedural justice was measured using two items (E11-1 and E11-2). Respondents rated their agreement with each statement on a five-point Likert scale ranging from 1 (“strongly disagree”) to 5 (“strongly agree”). The items were: “Police officers demonstrate a courteous approach when addressing community issues, exhibiting a high degree of responsibility and enthusiasm” (E11-1), and “The police make impartial judgments based on facts” (E11-2). The overall mean score for procedural justice was 3.930, with scores ranging from 1.5 to 5.

Distributive justice was assessed using a single item (E11-3): “Police officers maintain an impartial and fair approach toward every citizen during the course of their duties.” The same five-point scale was applied. The average score for this dimension was 3.757, with a range of 2–5.

##### 3.2.2.3 Police performance evaluations

In terms of police performance evaluations, this encompasses four specific variables: effectiveness of the stop and search (E9), perceptions of police integrity (E11-4, E11-5), police work capability (E12) and satisfaction with the police (E13).

To gage the effectiveness of the stop and search, each respondent was asked: “E9 What’s your evaluation of the effectiveness of police patrols and checks (such as crime prevention and combat)?” (1) Completely ineffective (2) Relatively ineffective (3) Average (4) Relatively effective (5) Very effective. This question ranks residents’ assessments of police stop-and-search effectiveness from lowest to highest, with options scored from 1 to 5. The overall average score for the effectiveness of stop and search is 4.171, with a range of 1–5.

To assess residents’ perceptions of police integrity, two items were administered. Respondents indicated their level of agreement on a five-point scale (1 = strongly disagree to 5 = strongly agree) with the following statements: (1) “E11-4 Police decisions are influenced by gifts, personal connections, or pressure from superiors,” and (2) “E11-5 Police officers often abuse their power.” These two items were combined to form a composite variable, labeled Perceived Police Integrity (reverse-coded). The mean score was 3.951, with the minimum and maximum values being 1 and 5, respectively.

Police work capability was assessed using five items (E12-1 through E12-5). Respondents were first presented with a general prompt: “Regarding your assessment of the police’s effectiveness in your community, do you agree with the following statements?” They then rated their agreement with each of the five statements on a five-point Likert scale (1 = strongly disagree to 5 = strongly agree). The items covered: (E12-1) Effective patrols and surveillance that enable timely identification and resolution of security risks; (E12-2) Commendable performance in maintaining public order, including mediating disputes and domestic conflicts; (E12-3) Prompt response to reports or calls for assistance; (E12-4) Efficient case handling coupled with sufficient emotional care and support for victims; and (E12-5) Strong performance in solving cases. The overall mean score for police work capability was 3.93 (range: 1.6–5).

To gage residents’ satisfaction with the police, each respondent was asked: “E13 How is the overall satisfaction rate of the police in your community (shequ)? (1) Very dissatisfied (2) Relatively dissatisfied (3) Neutral (4) Relatively satisfied (5) Very satisfied.” This question ranks residents’ overall satisfaction with the police from lowest to highest, with options scored from 1 to 5. Our statistical analysis reveals that the overall average for the indicator “residents” satisfaction with the police’ stands at 4.021, with a minimum value of 2 and a maximum value of 5.

##### 3.2.2.4 Legal socialization experience

Legal socialization experience contains 5 variables: whether residents sought police assistance (E1), whether one has been stopped and questioned (E8), frequency of encounters with police (E10), residents’ attitude toward the law (E15-1, E15-2), and residents’ endorsement of community norms (E15-3). The first three variables concern police–resident contact, and these contact experiences may shape residents’ legal attitudes, whereas the latter two capture their attitudes toward the law and social norms.

To ascertain whether residents sought police assistance, each respondent was asked: “E1 Have you ever called 110 to report the incident and seek help from the police? (1) Yes (2) No”. Yes was coded as 1 and No was coded as 0. Among them, 9.58% of respondents answered Yes, while 90.42% answered No.

To investigate whether one has been stopped and questioned, each respondent was asked: “E8 Have you ever been stopped by the police on the road and asked to show your ID card for questioning? (1) Yes (2) No”, yes is scored as 1, no is scored as 0. According to our statistics, we found that 28.70 percent of respondents answered “yes,” while 71.30 percent answered “no.”

To investigate the frequency of encounters with police, each respondent was asked: “E10 How often do you see the police in the following situations?” (“Would you say you hardly seen, rarely seen, occasionally seen, often seen or frequently seen?”). Frequency of encounters with police was measured across three dimensions. Respondents were asked: “E10-1 Within and around the community/residential area where you live, you can see police officers or security patrol personnel” and “E10-2 When you go out for activities (such as work, shopping, etc.), be able to see the police,” with options scored from 1 to 5. Through calculation, we determined that the overall average frequency of encounters with police is 3.567, with a minimum value of 1 and a maximum value of 5.

Then, to assess residents’ attitudes toward the law, each respondent was asked: “E15-2: For certain minor offenses (such as small-scale street trading), you believe a degree of tolerance and understanding may be granted” and “E15-3: One may adopt an attitude towards laws and regulations that “as long as you do not get caught, it’s not illegal?” The average score from these two questions was recorded as “residents’ attitude towards the law.” The two original questions in the questionnaire both measured residents’ negative attitudes toward the law. The response options were scored from 1 to 5 points, ranging from ‘Strongly Disagree’ to ‘Strongly Agree’. Under the original scoring scheme, higher scores indicated more negative attitudes toward the law, which differed in polarity from the other questions. Therefore, we have reversed the scoring, adjusting it so that responses ranging from ‘Strongly Disagree’ to ‘Strongly Agree’ are assigned scores from 5 to 1, respectively. The overall average score for residents’ attitude toward the law is 3.190, with a range of 1–5.

To assess residents’ endorsement of community norms, respondents were asked to rate their agreement with the following statement (E15-1): ‘Although uncivil behaviors in the community—such as spitting, failing to leash pets, and disorderly parking—do not violate the law, they should still be subject to moral condemnation or community norms.’ Responses were recorded on a five-point Likert scale ranging from 1 (‘strongly disagree’) to 5 (‘strongly agree’). The mean score for this item was 3.957, with scores ranging from 1 to 5.

##### 3.2.2.5 Perceptions of community environment

For environmental characteristics, three variables—social disorder, physical disorder, and community cohesion—were taken into account.

There are four dimensions for measuring physical disorder. Respondents rate the hygiene and safety conditions within and around their community (“Would you say this is highly inconsistent, not quite fitting, generally compliant, fairly consistent, very consistent?”). “C1-1: Public facilities within the community are frequently vandalized.”, “C1-2: Graffiti and unauthorized advertising are common within the community,” “C1-3: The common occurrence of litter being strewn about and vehicles parked haphazardly in the vicinity of the community” and “C1-4: The neighborhood is rather noisy.” From “highly inconsistent” to “very consistent,” these five options were scored sequentially from 1 to 5 points. The overall average was 2.541, with the minimum value being 1 and the maximum value being 5.

There are four dimensions for measuring social disorder. Respondents were asked about the frequency of the following phenomena occurring within and around their residential communities/estates (using the response options: “Would you say hardly ever, seldom, sometimes, often, or frequently?”): “C2-1: Residents in the community engage in verbal altercations and other conflicts,”. “C2-2: Suspicious strangers have been spotted within the community.”, “C2-3: Young people are gathering and loitering on the streets around the community,” “C2-4: There are often drunken disturbances in the neighborhood at night.”. The average score from these four questions is denoted as “social disorder.” From “never” to “very often,” we assigned scores sequentially from 1 to 5. Statistical analysis revealed an overall average score of 1.776 for social disorder, with responses ranging from 1 to 5.

There are five dimensions for measuring community cohesion. Respondents were asked to rate the following statements regarding community cohesion (“Would you say this is highly inconsistent, not quite fitting, generally compliant, fairly consistent or very consistent?”): “C3-1: In emergencies (such as an elderly person falling ill or a child going missing), neighbors are willing to help” “C3-2: Neighbors exchange festive greetings during traditional celebrations such as the Spring Festival and Mid-Autumn Festival, or jointly organize celebratory events.”, “C3-3: Neighborly interactions based on mutual trust (such as looking after elderly neighbors or children, or collecting parcels on behalf of others),” “C3-4: When disagreements arise between neighbors over everyday matters such as noise or the use of communal spaces, you tend to resolve issues through communication and negotiation rather than confrontation and conflict” “C3-5: Neighbors can reach a shared understanding on values such as mutual support within the community and the voluntary maintenance of public order.” Options ranging from “highly inconsistent” to “very consistent” were assigned scores from 1 to 5, respectively. Statistical analysis yielded an average community cohesion score of 3.814, with responses spanning the full 1 to 5 scale.

The descriptive statistics of dependent and independent variables are shown in Table 1.

**Table 1:** Descriptive statistics of dependent and independent variables.

Variable	Mean	Std. Dev.	Min.	Max.
Police legitimacy	4.004	0.532	1.000	5.000
Individual attributes				
Gender (female = 1)	0.473	0.499	0	1
Age	41.152	13.944	20	90
Marital status (married = 1)	0.707	0.455	0	1
Household registration status (local = 1)	0.435	0.496	0	1
Educational attainment	4.836	1.624	2	8
Personal monthly income	4.599	1.569	1	8
Length of residence	13.102	13.187	1	81
Perceived community socioeconomic status	3.162	0.788	1	5
Cantonese proficiency	3.887	1.366	1	5
Procedural justice and distributive justice				
Procedural justice	3.930	0.610	1.5	5
Distributive justice	3.757	0.760	2	5
Police performance evaluations				
Effectiveness of the stop and search	4.171	0.727	1	5
Perceptions of police integrity	3.951	0.819	1	5
Police work capability	3.927	0.537	1.6	5
Satisfaction with the police	4.021	0.587	2	5
Legal socialization experience				
Whether residents sought police assistance	0.096	0.294	0	1
Whether one has been stopped and questioned	0.287	0.452	0	1
Frequency of encounters with police	3.567	0.738	1	5
Residents’ attitude toward the law	3.190	0.898	1	5
Residents’ endorsement of community norms	3.957	0.741	1	5
Perceptions of community environment				
Physical disorder	2.541	0.908	1	5
Social disorder	1.776	0.672	1	5
Community cohesion	3.814	0.673	1	5

Besides, we assessed the reliability and validity of all scales used in the study. Internal consistency was evaluated using Cronbach’s *α*, with all values exceeding the conventional threshold of 0.7, indicating good reliability. For validity assessment, we conducted the Kaiser-Meyer-Olkin (KMO) test and Bartlett’s test of sphericity on each scale. The results showed KMO values ranging from 0.70 to 0.83, and Bartlett’s test yielded significant approximate *χ*^2^ values between 29.414 and 143.11, all of the indexes except for social disorder with *p*-value <0.001, confirming that generally, the scales possess good validity. Table 2 presents all multi-item indexes used in our study, their constituent items, and their reliability and validity statistics.

**Table 2:** Reliability and validity statistics for multi-item indexes.

Index	Constituent items	Number of items	Cronbach’s *α*	KMO	Bartlett’s *χ*^2^	*p*-value
Police legitimacy	E141, E142, E143, E144	4	0.77	0.7	143.11	<0.001
Police work capability	E12-1, E12-2, E12-3, E12-4, E12-5	5	0.82	0.82	68.243	<0.001
Physical disorder	C1-1, C1-2, C1-3, C1-4	4	0.87	0.8	29.414	<0.001
Social disorder	C2-1, C2-2, C2-3, C2-4	4	0.83	0.79	2.574	0.462
Community cohesion	C3-1, C3-2, C3-3, C3-4, C3-5	5	0.86	0.83	66.911	<0.001

### 3.3 Analytical strategy

This study employs a series of ordinary least squares (OLS) linear regression models to examine the factors influencing residents’ perceptions of police legitimacy. The analytical strategy involves estimating three models to progressively assess the contribution of different variable sets. Model 1 includes only individual attributes (e.g., age, gender, education, income). Model 2 adds variables capturing procedural/distributive justice, police performance evaluations, and legal socialization experience. Finally, Model 3 incorporates perceptions of community environment variables. For each of the three models, we estimated two parallel specifications: one using unstandardized coefficients (with all variables in their original metrics) and another using standardized coefficients (with the dependent variable and all continuous independent variables standardized; binary variables remained unstandardized to preserve interpretability as mean differences). This dual approach allows us to report unstandardized coefficients for interpreting effect sizes in original units, while standardized coefficients facilitate comparison of the relative importance of predictors across different variable types. Because respondents are nested within 72 communities, we report all models with community-level cluster-robust standard errors. All questionnaire data processing and model construction were completed using *R*.

It should be noted that community environment variables (physical disorder, social disorder, community cohesion) were measured at the individual perception level—each respondent reported their subjective evaluation of their neighborhood environment. We do not currently have independently aggregated neighborhood-level measures (e.g., official crime statistics, census-derived disadvantage indices) that would allow us to model true contextual effects. With only individual-level perceptual measures, a multilevel model would not yield meaningful distinctions between individual and community effects. We further calculated the within-community and between-community variances (Table 3). As can be seen, the within-community variances of the three environmental variables all exceed the between-community variances, which further confirms that multilevel modeling is not applicable to this survey data.

**Table 3:** Within-community and between-community variances of three environmental variables.

Variables	Within-community variance	Between-community variance
Physical disorder	0.498	0.332
Social disorder	0.290	0.164
Community cohesion	0.353	0.102

## 4 Results

This study employed linear regression models to examine the effects of individual attributes, procedural/distributive justice, police performance evaluations, legal socialization experience, and environment characteristics on the perceptions of police legitimacy. Three models were estimated, with Model 1 including only individual attributes, Model 2 adding procedural/distributive justice, police performance evaluations, and legal socialization experience, Model 3 further incorporating perceptions of community environment characteristics. All models were estimated using a sample of *N* = 1899. With VIF values for the independent variables ranging from 1.051 to 2.262, multicollinearity does not pose a threat to the reliability of the estimates. Table 4 presents the models result, including both unstandardized (coef.) and standardized (*β*) coefficients.

**Table 4:** Model results.

Variables	Model 1		Model 2		Model 3		
	*β*	std. *β*	*β*	std. *β*	*β*	std. *β*	VIF
Individual attributes							
Gender (female = 1)	0.036	0.068	−0.002	−0.003	0.002	0.003	1.146
Age	0.002	0.051	0.001	0.033	0.001	0.031	2.622
Marital status (married = 1)	−0.061^+^	−0.115	−0.065^*^	−0.123	−0.068^**^	−0128	1.506
Household registration status (local = 1)	0.075^+^	0.140	0.072^*^	0.135	0.067^*^	0.126	1.995
Educational attainment	−0.012	−0.038	0.0003	0.0008	0.0003	0.001	2.135
Personal monthly income	0.003	0.010	−0.005	−0.013	−0.003	−0.009	1.277
Length of residence	0.001	0.031	−0.001	−0.021	−0.001	−0.020	1.547
Perceived community socioeconomic status	−0.004	−0.006	−0.049^**^	−0.072	−0.053^**^	−0.079	1.261
Cantonese proficiency	0.009	0.017	0.004	0.007	0.002	0.003	1.620
Procedural justice and distributive justice							
Procedural justice			0.048^*^	0.055	0.040^+^	0.046	1.997
Distributive justice			0.061^**^	0.087	0.068^***^	0.096	1.555
Police performance evaluations							
Effectiveness of the stop and search			0.085^***^	0.116	0.083^***^	0.113	1.364
Perceptions of police integrity			0.026	0.039	0.024	0.037	1.105
Police work capability			0.282^***^	0.284	0.275^***^	0.278	2.121
Satisfaction with the police			0.173^***^	0.191	0.179^***^	0.197	1.657
Legal socialization experience							
Whether residents sought police assistance			0.019	0.035	0.013	0.024	1.051
Whether one has been stopped and questioned			−0.083^**^	−0.155	−0.072^**^	−0.136	1.197
Frequency of encounters with police			0.039^+^	0.054	0.026	0.036	1.439
Residents’ attitude toward the law			−0.067^***^	−0.114	−0.068^***^	−0.115	1.093
Residents’ endorsement of community norms			0.020	0.028	0.014	0.020	1.223
Perceptions of community environment							
Physical disorder					−0.029	−0.049	1.521
Social disorder					0.023	0.028	1.486
Community cohesion					0.047^*^	0.060	1.267
*N*	1899		1899		1899		
Adjusted *R*^2^	0.013		0.396		0.399		

Significance levels: ^***^p < 0.001, ^**^p < 0.01, ^*^p < 0.05, ^+^*p* < 0.1; *β* = unstandardized coefficient; std. *β* = standardized coefficient. Community-level cluster-robust standard errors are estimated.

Model 1 explained only 1.3% of the variance in the outcome (Adjusted *R*^2^ = 0.013), indicating that individual demographic and socioeconomic variables alone had limited explanatory power. With the inclusion of variables of procedural/distributive justice, police performance evaluations, and legal socialization experience, Model 2’s explanatory power increased substantially (Adjusted *R*^2^ = 0.396), suggesting that variables such as perceptions and experiences with police are critical predictors. Model 3, which added residents’ perceptions of community environment, showed a slight improvement in fit (Adjusted *R*^2^ = 0.399).

As shown in Model 3, in terms of individual attributes, married or not (*β* = −0.068, *p* < 0.01) and perceived community socioeconomic status (*β* = −0.053, *p* < 0.01) are significantly negatively associated with perceived police legitimacy. This indicates that married residents and those with higher perceived socioeconomic status tend to report lower evaluations of police legitimacy. Local hukou status (*β* = 0.067, *p* < 0.05) is significantly positively associated with perceived police legitimacy, indicating that local residents tend to report higher evaluations of police legitimacy.

Both procedural justice and distributive justice are significantly positively associated with police legitimacy perceptions, with distributive justice (std. *β* = 0.096, *p* < 0.001) exerting a stronger association than procedural justice (std. *β* = 0.046, *p* < 0.1).

As for police performance evaluations, effectiveness of the stop and search, police work capability and satisfaction with the police are all significantly positively associated with police legitimacy perceptions. Of these, the strongest association is police work capability (std. *β* = 0.278, *p* < 0.001), followed by satisfaction with the police (std. *β* = 0.197, *p* < 0.001), and effectiveness of the stop and search (std. *β* = 0.113, *p* < 0.001), and perceptions of police integrity (std. *β* = 0.037, *p* < 0.05). Perceptions of police integrity were positively but non-significantly associated with perceptions of police legitimacy (std. *β* = 0.037, *p* ≈ 0.143), indicating a very small and statistically unreliable effect. It should be noted that these variables are built from respondents’ subjective assessments of police performance, not from objective performance indicators.

Regarding legal socialization experience, whether one has been stopped and questioned (*β* = −0.072, *p* < 0.01) and residents’ attitude toward the law (*β* = −0.068, *p* < 0.001) are significantly negatively associated with the evaluation of police legitimacy. This implies that residents who have been previously stopped and questioned, as well as those with stricter legal attitudes, tend to report lower evaluations of police legitimacy.

In terms of environment characteristics, physical disorder shows a negative but statistically non-significant association (*β* = −0.029, *p* ≈ 0.104), while community cohesion has a significant positive association (*β* = 0.047, *p* < 0.05). These results suggest that stronger community cohesion is associated with higher evaluations of police legitimacy, while the negative association between physical disorder and police legitimacy, although in the expected direction, is not statistically supported. The coefficient for social disorder is not statistically significant. This null result is more likely a consequence of measurement limitations than evidence that social disorder is genuinely unrelated to perceived police legitimacy. We therefore do not treat it as substantive evidence of no effect.

## 5 Discussion and conclusions

This study set out to address key gaps in the literature on police legitimacy by integrating individual attributes, procedural/distributive justice, police performance evaluations, legal socialization experience, and perceived community environment within the Chinese context. Drawing on original survey data from 72 diverse communities in Guangzhou, China, our findings provide robust empirical evidence that perceptions of police legitimacy are shaped by a complex interplay of these five dimensions, with interactional experiences emerging as the most potent driver. Below, we discuss the theoretical and policy implications of our key findings, situate them within the broader literature, and identify directions for future research.

The most striking finding of our study is the overwhelming explanatory power of police–resident interactional factors. The inclusion of these variables increased the model’s explanatory power from a mere 1.3% to nearly 40% (Adjusted *R*^2^ = 0.396), underscoring that how residents experience the police in their daily lives is paramount. Among these, police work capability emerged as the strongest positive predictor of legitimacy (std. *β* = 0.278, *p* < 0.001), followed by overall satisfaction with the police (std. *β* = 0.197, *p* < 0.001), and perceived effectiveness of the stop and research (std. *β* = 0.113, *p* < 0.001). This hierarchy of effects suggests that in Guangzhou—a megacity striving for modern, efficient governance—the public’s evaluation of police legitimacy is heavily associated with the police’s perceived competence, responsiveness, and ability to deliver tangible security outcomes.

These findings both align with and complicate the procedural justice paradigm. While Tyler (2006) process-based model has long held that procedural fairness is the primary driver of legitimacy, our results indicate that instrumental performance considerations may carry substantial independent weight in the Chinese urban context. This is consistent with prior research in non-Western settings suggesting that citizens in rapidly modernizing societies may prioritize effective crime control and visible policing alongside—or even ahead of—procedural fairness (Sun et al., 2018; Cao, 2022). However, it would be premature to conclude that procedural justice is unimportant. Both procedural and distributive justice exhibited significant associations (std. *β* = 0.046 and 0.096, respectively), confirming that fair treatment and equitable outcomes remain meaningful contributors to legitimacy even when performance evaluations are accounted for. Rather than viewing instrumental and normative motives as competing alternatives, our findings suggest they operate as complementary pathways, echoing recent arguments by Na et al. (2023) in the Korean context. This complementarity implies that effective policing strategies must attend to both what police achieve (crime control, responsiveness) and how they achieve it (fairness, respect, transparency).

The negative association of being stopped and questioned by police (std. *β* = −0.136, *p* < 0.01) resonates with global concerns about the legitimacy costs of proactive enforcement tactics. Residents who had experienced police stops reported lower legitimacy, even when controlling for their evaluations of stop effectiveness at the aggregate level. This finding suggests that the individual experience of being subjected to police scrutiny—particularly if perceived as arbitrary, disrespectful, or unjustified—can erode trust irrespective of overall performance perceptions. This mirrors findings from Western contexts (Brunson and Miller, 2006; Bradford et al., 2025) and highlights a critical policy challenge: while proactive policing tactics such as stop-and-search are intended to deter crime, their implementation can inadvertently undermine the very legitimacy they seek to bolster if not conducted with clear justification, procedural fairness, and transparency.

Our findings on individual attributes challenge some conventional assumptions while confirming others. Contrary to the often-assumed positive link between socioeconomic status and institutional trust, we found that perceived community socioeconomic status was negatively associated with police legitimacy (std. *β* = −0.079, *p* < 0.01). Similarly, married individuals reported lower legitimacy ratings compared to their unmarried counterparts (std. *β* = −0.128, *p* < 0.01). One plausible interpretation is that individuals with greater social and economic capital—and those embedded in more stable family structures—may possess a stronger sense of rights and a more critical orientation toward state authority, making them less deferential and more likely to notice or be troubled by procedural shortcomings (Weitzer and Tuch, 1999; Hawdon, 2003). This aligns with the “legitimacy paradox” identified in prior research, wherein higher social standing fosters greater scrutiny rather than greater trust.

Notably, local hukou holders reported significantly higher legitimacy than non-local residents (std. *β* = 0.126, *p* < 0.05). This finding reinforces the idea that institutional insiders—those with formal belonging to the city—tend to view its authorities more favorably than outsiders, a pattern consistent with prior Chinese studies (Wu et al., 2016; Wang and Sun, 2020). The hukou system, however, is not merely an individual attribute but a macro-level institution that allocates rights and entitlements, and it may affect legitimacy judgments through several routes: material (differential access to public services shapes whether institutions are experienced as fair), experiential (non-local residents may face more identity checks, making their police encounters more likely to be involuntary and perceived as procedurally unjust), and identity-based (the household registration system shapes how residents understand their position in the city). These multiple pathways help explain why the effect size of hukou was relatively modest compared to interactional factors: while institutional stratification matters, its influence is mediated or overshadowed by direct experiences with police. The mixed findings in the broader hukou literature—some studies finding lower trust among migrants, others finding higher endorsement (Sun et al., 2013)—underscore that the hukou-legitimacy relationship is not deterministic but contingent on individual expectations, perceived social status, and the specific dimension of trust under examination. Our results contribute to this debate by demonstrating that hukou retains a significant independent effect even when interactional experiences and neighborhood conditions are controlled, though its explanatory power is limited relative to performance evaluations.

Interestingly, Cantonese proficiency—a culturally salient marker of local identity in Guangzhou—did not emerge as a significant predictor in our final model. This may reflect the widespread prevalence of Mandarin Chinese in formal institutional settings, such that language proficiency does not substantially shape police interactions or legitimacy perceptions in this context. Alternatively, it may suggest that institutional belonging (hukou) rather than cultural assimilation is the more consequential axis of stratification in shaping police trust.

Our findings confirm that neighborhood environmental characteristics provide a crucial context for legitimacy evaluations, even after accounting for individual attributes and interactional experiences. Community cohesion emerged as a significant positive predictor (std. *β* = 0.060, *p* < 0.05), indicating that residents living in neighborhoods with stronger social bonds and mutual trust tend to view police more favorably. This aligns with collective efficacy theory (Sampson et al., 1997) and prior Chinese research Liu et al. (2020), suggesting that cohesive communities may foster a more collaborative relationship with local law enforcement, creating a virtuous cycle of trust and cooperation. Conversely, physical disorder is associated with lower perceived legitimacy (std. *β* = −0.049, *p* ≈ 0.104). This finding extends prior work by Zhao et al. (2014), who found that objective disorder crime did not directly affect police performance perceptions but operated indirectly through residents’ subjective perceptions. Our results suggest that perceived physical deterioration—vandalism, graffiti, litter—directly undermines legitimacy evaluations, possibly because residents interpret such signs as indicative of inadequate policing or state neglect.

Interestingly, social disorder—measured by conflicts, suspicious strangers, and public disturbances—did not reach statistical significance in our final model. This divergence between physical and social disorder merits attention. One possibility is that physical disorder is more visually salient and directly attributable to police inaction, whereas social disorder may be perceived as reflecting broader socioeconomic conditions beyond police control. Alternatively, our measure of social disorder may capture lower-frequency events that residents encounter less consistently than the physical signs of neighborhood decay. This nuanced finding underscores the importance of disaggregating environmental characteristics rather than treating them as a unitary construct.

These findings have profound implications for policing policy in China and other non-Western megacities. First, they strongly argue for a decisive shift away from campaign-style, reactive policing toward a model of routinized, law-based enforcement that prioritizes the quality of everyday interactions. Police training and evaluation should be reoriented to emphasize not just crime-fighting metrics but also the principles of procedural justice: treating citizens with dignity, making fair and transparent decisions, and demonstrating genuine helpfulness. Second, the negative association of police stops underscores the need for clear guidelines, officer accountability, and community dialog around proactive policing tactics to ensure they build, rather than break, trust. Third, the importance of community cohesion suggests that police should actively partner with neighborhood organizations and residents to strengthen local social fabric, recognizing that community health and police legitimacy are mutually reinforcing.

Several limitations of this study should be acknowledged. First, although our environmental variables captured residents’ subjective perceptions of neighborhood conditions, they were measured at the individual level rather than through independently aggregated neighborhood-level indicators (e.g., official crime statistics, census-derived disadvantage indices). While we confirmed that within-community variance exceeded between-community variance—which justified our OLS approach—future research would benefit from multi-level data structures that allow for true contextual effects modeling, integrating objective neighborhood measures with subjective perceptions to provide a more complete picture of how environmental conditions shape legitimacy. Second, our measure of police legitimacy, while grounded in established scales, relied on self-reported attitudes that may be subject to social desirability bias (Zhang et al., 2007). Future research could incorporate behavioral measures of cooperation or compliance alongside attitudinal measures to triangulate legitimacy perceptions and mitigate potential response biases. Third, distributive justice was measured with a single item, so its reliability cannot be estimated and measurement error cannot be quantified. Because measurement error attenuates estimated associations, the coefficient for distributive justice should be regarded as a lower bound. Fourth, given the cross-sectional design, reverse causality cannot be ruled out: residents with lower legitimacy perceptions may be more likely to interpret police stops as unjustified. Longitudinal or experimental designs are therefore needed to establish causal directions. Fifth, our findings are based on an empirical study of residents in Guangzhou, and whether this result generalizes to other Chinese cities still requires further empirical investigation.

In conclusion, this study demonstrates that a comprehensive approach is essential for understanding police legitimacy in complex urban settings like Guangzhou, China. While individual demographics provide a baseline, it is the lived experience of interacting with the police and the broader social context of one’s neighborhood that ultimately shape public trust. By foregrounding these interactional and contextual dynamics, our research provides a more nuanced and empirically grounded foundation for developing differentiated, effective, and legitimacy-enhancing community-governance strategies in China.

## Data Availability

The data presented in this study are available on request from the corresponding authors.
