# Supplementary material for: Perceptions of police legitimacy and its influencing factors: evidence from Guangzhou, China
Source: Front Psychol. 2026 Oct 26;17:1903028. doi: 10.3389/fpsyg.2026.1903028 (PMC13745329; doi:10.3389/fpsyg.2026.1903028)
Supplement: Supplementary file 1 [file Table_1.DOCX]

# Appendix A：

Gender：

A1: Your gender is: (1) Male (2) Female

Age:

A2: May I ask your date of birth: ________ (year) ________(month)

Marital status:

A3 Your marital status is:

(1) Unmarried (2) Married (3) Divorced or widowed

Whether the resident hold local household registration?

A4 Your household registration is in _________ city, _________ province.

Educational attainment:

A5 What is your educational attainment?

(1) No formal education

(2) Primary school

(3) Junior high school

(4) High school

(5) Secondary vocational school/technical secondary school/vocational high school;

(6) junior college;

(7) bachelor's degree;

(8) Postgraduate degree or higher.

Personal monthly income:

A8 Your personal average monthly income is ______ (including salary, bonus, dividend, pension or other part-time income) :

(1) Under 1,000 yuan (2) 1,000-1,999 yuan (3) 2,000-2,999 yuan (4) 3,000-4,999 yuan

(5) 5,000-7,999 yuan (6) 8,000-9,999 yuan (7) 10,000-20,000 yuan (8) Over 20,000 yuan.

Length of residence:

A11 When did you start living in this community/neighborhood: ______ year(s) ______ month(s).

Perceived community socioeconomic status:

A14: Please rate your community's standing in Guangzhou. (1) Low (2) relatively low (3) average (4) relatively high (5) high

Cantonese proficiency:

A17 Can you speak Cantonese?

(1) Not at all.

(2) Can listen a little but not speak.

(3) Can speak a little.

(4) Basically can.

(5) Very fluent.

Physical disorder:

C1: Please evaluate and score the following aspects of hygiene and safety within and around the community/residential area：

|  | | highly inconsistent | Not quite fitting | Generally compliant | Fairly consistent | Very consistent |
| --- | --- | --- | --- | --- | --- | --- |
| **C1-1** | Please evaluate and score the following aspects of hygiene and safety within and around the community/residential area. | （1） | （2） | （3） | （4） | （5） |
| **C1-2** | Graffiti and unauthorised advertising posters are commonplace within the community. | （1） | （2） | （3） | （4） | （5） |
| **C1-3** | The common occurrence of litter being strewn about and vehicles parked haphazardly in the vicinity of the community. | （1） | （2） | （3） | （4） | （5） |
| **C1-4** | The neighbourhood is rather noisy. | （1） | （2） | （3） | （4） | （5） |

Social disorder:

Frequency of occurrence of the following phenomena within and around their residential communities/estates:

|  |  | Hardly ever | Seldom | Sometimes | Often | Frequently |  |
| --- | --- | --- | --- | --- | --- | --- | --- |
| **C2-1** | Residents in the community engage in verbal altercations and other conflicts. | （1） | （2） | （3） | （4） | （5） |  |
| **C2-2** | Suspicious strangers have been spotted within the community. | （1） | （2） | （3） | （4） | （5） |  |
| **C2-3** | Young people are gathering and loitering on the streets around the community. | （1） | （2） | （3） | （4） | （5） |  |
| **C2-4** | There are often drunken disturbances in the neighbourhood at night. | （1） | （2） | （3） | （4） | （5） |  |

Community cohesion:

C3: Please rate the following description of community cohesion:

C3-1: In emergencies (such as an elderly person falling ill or a child going missing), neighbours are willing to help.

C3-2: Neighbours exchange festive greetings during traditional celebrations such as the Spring Festival and Mid-Autumn Festival, or jointly organise celebratory events.

C3-3: Neighbourly interactions based on mutual trust (such as looking after elderly neighbours or children, or collecting parcels on behalf of others)

C3-4: When disagreements arise between neighbours over everyday matters such as noise or the use of communal spaces, you tend to resolve issues through communication and negotiation rather than confrontation and conflict.

C3-5: Neighbours can reach a shared understanding on values such as mutual support within the community and the voluntary maintenance of public order.

Options ranging from one point to five points: highly inconsistent, Not quite fitting, Generally compliant, Fairly consistent, Very consistent.

|  |  | Highly inconsistent | Not quite fitting | Generally compliant | Fairly consistent | Very consistent |  |
| --- | --- | --- | --- | --- | --- | --- | --- |
| **C3-1** | In emergencies (such as an elderly person falling ill or a child going missing), neighbours are willing tohelp. | （1） | （2） | （3） | （4） | （5） |  |
| **C3-2** | Neighbours exchange festive greetings during traditional celebrations such as the Spring Festival and Mid-Autumn Festival, or jointly organise celebratory events. | （1） | （2） | （3） | （4） | （5） |  |
| **C3-3** | Neighbourly interactions based on mutual trust (such as looking after elderly neighbours or children, or collecting parcels on behalf of others) | （1） | （2） | （3） | （4） | （5） |  |
| **C3-4** | When disagreements arise between neighbours over everyday matters such as noise or the use of communal spaces, you tend to resolve issues through communication and negotiation rather than confrontation and conflict. | （1） | （2） | （3） | （4） | （5） |  |
| **C3-5** | Neighbours can reach a shared understanding on values such as mutual support within the community and the voluntary maintenance of public order. | （1） | （2） | （3） | （4） | （5） |  |

Whether residents sought police assistance:

E1: Have you ever called 110 to report the incident and seek help from the police?

(1)Yes

(2) None (jump to E8)

Whether one has been stopped and questioned:

E8: Have you ever been stopped by the police on the road and asked to show your ID card for questioning?

(1)Yes

(2) None

Effectiveness of the stop and search:

E9: What's your evaluation of the effectiveness of police patrols and checks (such as crime prevention and combat)?

(1) Completely ineffective (2) Relatively ineffective (3) Average (4) relatively effective (5) very effective

Frequency of encounters with police:

E10 How often do you see the police in the following situations?

|  |  | Hardly seen | Rarely seen | Occasionally seen | Often seen | Frequently seen |  |
| --- | --- | --- | --- | --- | --- | --- | --- |
| **E10-1** | Within and around the community/residential area where you live, you can see police officers or security patrol personnel. | （1） | （2） | （3） | （4） | （5） |  |
| **E10-2** | When you go out for activities (such as work, shopping, etc.), be able to see the police. | （1） | （2） | （3） | （4） | （5） |  |

**Procedural / distributive justice**:

E11 Regarding your evaluation of the performance and professional ethics of the police officers near the community/residential area, do you agree with the following viewpoints?

|  |  | Strongly disagree | Not quite agree | Neither agree nor disagree | Relatively agree | Strongly agree |  |
| --- | --- | --- | --- | --- | --- | --- | --- |
| **E11-1** | Police officers demonstrate a courteous approach when addressing community issues, exhibiting a high degree of responsibility and enthusiasm. | （1） | （2） | （3） | （4） | （5） |  |
| **E11-2** | The police make impartial judgements based on the facts. | （1） | （2） | （3） | （4） | （5） |  |
| **E11-3** | Police officers maintain an impartial and fair approach towards every citizen during the course of their duties. | （1） | （2） | （3） | （4） | （5） |  |

Perceptions of police integrity:

E11 Regarding your evaluation of the performance and police integrity of the police officers near the community/residential area, do you agree with the following viewpoints?

|  |  | Strongly disagree | Not quite agree | Neither agree nor disagree | Relatively agree | Strongly agree |  |
| --- | --- | --- | --- | --- | --- | --- | --- |
| **E11-4** | Police decisions are influenced by gifts, personal connections, or pressure from superiors. | （1） | （2） | （3） | （4） | （5） |  |
| **E1-15** | Police officers often abuse their power. | （1） | （2） | （3） | （4） | （5） |  |

Police work capability:

E12: Regarding your assessment of the police's effectiveness in your neighbourhood/community, do you agree with the following statements?

|  |  | Strongly disagree | Not quite fitting | Neither agree nor disagree | Relatively agree | Strongly agree |  |
| --- | --- | --- | --- | --- | --- | --- | --- |
| **E12-1** | Effective patrols and surveillance that enable timely identification and resolution of security risks | （1） | （2） | （3） | （4） | （5） |  |
| **E12-2** | Commendable performance in maintaining public order, including mediating disputes and domestic conflicts. | （1） | （2） | （3） | （4） | （5） |  |
| **E12-3** | Prompt response to reports or calls for assistance. | （1） | （2） | （3） | （4） | （5） |  |
| **E12-4** | Efficient case handling coupled with sufficient emotional care and support for victims. | （1） | （2） | （3） | （4） | （5） |  |
| **E12-5** | Strong performance in solving cases. | （1） | （2） | （3） | （4） | （5） |  |

Satisfaction with the police:

E13: How is the overall satisfaction rate of the police in your community/neighborhood?

(1) Very dissatisfied (2) Relatively dissatisfied (3) Neutral (4) Relatively satisfied (5) Very satisfied

Police legitimacy :

E14 Regarding your assessment of the legitimacy of the police near the community/residential area, do you agree with the following viewpoints?

|  |  | Strongly disagree | Not quite fitting | Neither agree nor disagree | Relatively agree | Strongly agree |  |
| --- | --- | --- | --- | --- | --- | --- | --- |
| **E14-1** | I hold the police in high regard. | （1） | （2） | （3） | （4） | （5） |  |
| **E14-2** | People should support the work of the police. | （1） | （2） | （3） | （4） | （5） |  |
| **E14-3** | I would comply with police decisions during law enforcement even if I did not fully agree with them. | （1） | （2） | （3） | （4） | （5） |  |
| **E14-4** | The sense of right and wrong upheld by the police in handling cases aligns with my own values. | （1） | （2） | （3） | （4） | （5） |  |

Residents’ attitude towards the law:

E15 To what extent do you agree or disagree with the following statements? (Attitudes towards the law)

|  |  | Strongly disagree | Not quite agree | Neither agree nor disagree | Relatively agree | Strongly agree |  |
| --- | --- | --- | --- | --- | --- | --- | --- |
| **E15-1** | Although uncivil behaviors in the community—such as spitting, failing to leash pets, and disorderly parking—do not violate the law, they should still be subject to moral condemnation or community norms. | （1） | （2） | （3） | （4） | （5） |  |
| **E15-2** | For certain minor offences (such as small-scale street trading), you believe a degree of tolerance and understanding may be granted. | （1） | （2） | （3） | （4） | （5） |  |
| **E15-3** | One may adopt an attitude towards laws and regulations that “as long as you don't get caught, it's not illegal. | （1） | （2） | （3） | （4） | （5） |  |
